# Spatiotemporal Empirical Mode Decomposition of Resting-State fMRI Signals: Application to Global Signal Regression

**DOI:** 10.3389/fnins.2019.00736

**Published:** 2019-07-23

**Authors:** Narges Moradi, Mehdy Dousty, Roberto C. Sotero

**Affiliations:** ^1^Biomedical Engineering Graduate Program, University of Calgary, Calgary, AB, Canada; ^2^Hotchkiss Brain Institute, University of Calgary, Calgary, AB, Canada; ^3^Computational Neurophysics Lab, Department of Radiology, University of Calgary, Calgary, AB, Canada; ^4^Institute of Biomaterials and Biomedical Engineering, University of Toronto, Toronto, ON, Canada; ^5^KITE, Toronto Rehab, University Health Network, Toronto, ON, Canada

**Keywords:** resting-state functional connectivity MRI, global Signal, fMRI, empirical mode decomposition, spatial intrinsic mode function, temporal intrinsic mode function, low-pass filtering

## Abstract

Resting-state functional connectivity MRI (rs-fcMRI) is a common method for mapping functional brain networks. However, estimation of these networks is affected by the presence of a common global systemic noise, or global signal (GS). Previous studies have shown that the common preprocessing steps of removing the GS may create spurious correlations between brain regions. In this paper, we decompose fMRI signals into 5 spatial and 3 temporal intrinsic mode functions (SIMF and TIMF, respectively) by means of the empirical mode decomposition (EMD), which is an adaptive data-driven method widely used to analyze non-linear and non-stationary phenomena. For each SIMF, functional connectivity matrices were computed by means of Pearson correlation between TIMFs of different brain areas. Thus, instead of a single connectivity matrix, we obtained 5 × 3 = 15 functional connectivity matrices. Given the high correlation and global efficiency values of the connectivity matrices related to the low spatial maps (SIMF3, SIMF4, and SIMF5), our results suggest that these maps can be considered as spatial global signal masks. Thus, by summing up the first two SIMFs extracted from the fMRI signals, we have automatically excluded the GS which is now voxel-specific. We compared the performance of our method with the conventional GS regression and to the results when the GS was not removed. While the correlation pattern identified by the other methods suffers from a low level of precision in identifying the correct brain network connectivity, our approach demonstrated expected connectivity patterns for the default mode network and task-positive network.

## 1. Introduction

Resting-state functional connectivity MRI (rs-fcMRI) has considerable potential for mapping functional brain networks (Biswal et al., 1995; Kandel et al., 2000; De Luca et al., 2006; Fox et al., 2006; Shmuel and Leopold, 2008; Friston, 2011). This mapping, which reveals the brain's functional architecture and operational principles (Kandel et al., 2000; Friston, 2011), can be used for early detection of brain connectivity pathologies in neuropsychiatric patients (Erdoğan et al., 2016). However, the presence of broadly shared synchronous fluctuations, termed as the global signal (GS) in Blood Oxygen Level Dependent (BOLD) responses, is a significant problem for fcMRI analysis. Its presence is problematic as it is of unknown origin (Damoiseaux et al., 2006; Fox et al., 2009; Erdoğan et al., 2016). Therefore, effective removal of GS has become an important step in data preprocessing and must be done prior to fcMRI analysis. GS is generally defined as the average of the BOLD signals over the whole brain (Zarahn et al., 1997; Fox et al., 2009; Liu et al., 2017) and can be computed from the raw images or after some preprocessing steps (Liu et al., 2017). The average-based GS is typically called conventional GS (or static GS (SGS) Erdoğan et al., 2016).

Application of SGS regression (SGSR) was at first just limited to task-related fMRI imaging (Zarahn et al., 1997; Macey et al., 2004). More recently, SGSR usage has received more attention in the analysis of resting-state fMRI than in task-related fMRI studies (Liu et al., 2017). Some studies suggest that application of SGSR improves the functional specificity of resting-state correlation maps and helps to remove non-neuronal sources of global variance like respiration and movement (Fox and Raichle, 2007; Fox et al., 2009; Liu et al., 2017). However, other studies found that these improvements are limited to systems that would exhibit only positive correlations with the specific selected seeds (Fox et al., 2009; Weissenbacher et al., 2009). On the other hand, many studies have shown that the common preprocessing steps of removing GS via a general linear model can create correlations between regions that may never have existed (Murphy et al., 2009; Anderson et al., 2010; Saad et al., 2012; Murphy and Fox, 2017), which results in spurious fcMRI values. Moreover, it has been shown that SGSR do not consider the brain's spatial heterogeneities and biases correlations in different regions of the brain (Saad et al., 2012). Accordingly, the extracted correlation maps are known to present artifacts and do not reflect underlying neurological properties (Murphy et al., 2009; Anderson et al., 2010; Saad et al., 2012; Murphy and Fox, 2017). Therefore, regressing out GS is under debate as its removal by applying current approaches may introduce artifacts into the fMRI data or cause the loss of important neuronal components (Murphy et al., 2009; Anderson et al., 2010; Saad et al., 2012; Murphy and Fox, 2017). These concerns about the GSR methods and the need for accurate brain functional connectivity maps motivate the need to develop new methods for dealing with GS. Moreover, it has been shown that GS has a variety of sources with different spatial distributions which are voxel-specific. Accordingly, it is desirable to use a new method that works selectively and is able to identify and remove the spatially specific GS for each voxel or region (Saad et al., 2012; Tong and Frederick, 2014; Chang et al., 2016; Power et al., 2017; Turchi et al., 2018), and also produce known connectivity patterns in networks such as the default mode network and task-positive network (Fox et al., 2009; Erdoğan et al., 2016), thus avoiding the creation of spurious correlations.

In addition to GS, in fMRI studies, BOLD signal is low-pass filtered (<0.1 Hz) during the preprocessing procedure to be sure that the effects of the high frequency physiological noises are removed from the data (Boubela et al., 2013; Brooks et al., 2013; Liu et al., 2017). This is because, physiological noises which are mainly cardiac and respiratory, are spatially widespread and have cycles located prominently at the frequency range of 0.1–2.5 Hz. It is indicated that, among different noise-removal methods (such as band-pass filtering and Independent component analysis), EMD based methods facilitate the noise removal from fMRI data. In EMD-based methods, IMFs with specific frequency bands are identified and removed from fMRI data to enhance the functional sensitivity of the data (Typically the first and second IMFs which have the highest frequency bands among all IMFs are considered as a noise) (Lin et al., 2016). However, removing the whole high-frequency data from fMRI time series is controversial, as smoothing the signals via low-pass filtering decreases the signal to noise ratio by smoothing the peaks and amplifying the noise (Brooks et al., 2013). In fact, it has been shown that filtering high frequency modes may also remove the signal of interest that contains similar frequencies. The main reason is that the TR time for sampling fMRI data is too low to distinguish the high frequency components and causes signal's frequencies being aliased that can not be separated by temporal filtering (Brooks et al., 2013). Furthermore, even using very high sampling rate (TR < 0.4 s) to detect the high frequency modes may cause losing information of neuronal activation in high frequencies by filtering high frequency modes (Tagliazucchi et al., 2011, 2012; Boubela et al., 2013). Accordingly, in resting-state studies, we cannot do the band-pass filtering through previous methods as the brain dynamics in all frequency bands needs to be investigated. Therefore, we need a method that can remove the physiological noises more specifically from BOLD signal.

There are several signal processing methods, such as Fourier transform (Gallagher et al., 2008), Wavelet transform (Yves, 1993), spatial and temporal Blind source separation (Comon and Jutten, 2010), and the EMD (Huang et al., 1998). However, all of the mentioned method except EMD require predefined basis function or some prior knowledge to decompose the signal. Considering the fact that real-world signals including fMRI signals are non-linear and non-stationary data and do not perfectly obey our assumption, EMD method would be the best method to apply, as it does not need any basis functions and parameters that need to be adjusted such as wavelet type in wavelet transform or informed separation ideas in Blind source separation method (Liutkus et al., 2013; Riffi et al., 2014; He et al., 2017). EMD is a computationally efficient method that can adaptively decompose any non-linear and non-stationary signals into Intrinsic mode functions (IMF) and obtain meaningful frequencies estimation (Huang et al., 1998; Mandic et al., 2008; Riffi et al., 2014; He et al., 2017).

In this paper, we define an adaptive global signal regression (AGSR) by performing a spatiotemporal decomposition of the fMRI time series through EMD-based methods. The GS which is computed using this method is voxel-specific and depends on brain regions' heterogeneity.

Additionally, we show that by applying AGSR, we do not need the traditional low-pass filtering methods as the proposed method exhibits the potential to adaptively remove the physiological noises from high temporal frequency modes of fMRI time series, that are shared in whole brain regions. Therefore, AGSR method, besides removing the GS, helps to eliminate the high frequency physiological noises without needing to perform the low-pass filtering step separately.

In AGSR method, We do not use the Multidimensional EMD approach as it requires great runtime and cannot decompose a multidimensional signal into multidimensional components (Wu et al., 2009; Riffi et al., 2014; He et al., 2017). Consequently, in this paper, two EMD-based methods are used sequentially to decompose the fMRI signals adaptatively and voxel-specifically. We acquired the Spatial and Temporal Intrinsic Mode Functions (SIMF and TIMF, respectively) of fMRI data by applying FATEMD (Riffi et al., 2014) and ICEEMDAN (Colominas et al., 2014) methods, respectively (Huang et al., 1998; Mandic et al., 2008). It has been shown that applying EMD-based methods on fMRI data separate inherent brain oscillations and fundamental modes embedded in BOLD signal. Each of these oscillations occupies a unique frequency band and can be used to investigate the frequency characteristics in resting-state brain networks (McGonigle et al., 2010; Zheng et al., 2010; Niazy et al., 2011; Song et al., 2014, 2015; Qian et al., 2015; Lin et al., 2016; Cordes et al., 2018).

To explore the frequency characteristics of the brain networks, first, we obtain the average functional connectivity matrices for different TIMFs of each SIMFs over all subjects. Functional connectivity was computed using pearsons' coefficient between the peak voxels of each brain regions included in the AAL 116 atlas (Tzourio-Mazoyer et al., 2002).

We then compute the efficiency (Fair et al., 2007; Rubinov and Sporns, 2009; Cohen and D'Esposito, 2016) of the brain network of different spatiotemporal IMFs, which is used as a measure of integration. Integration values are used to identify the GS, since GS is defined as a synchronous fluctuation which is shared among all brain regions that makes it being highly integrated in the whole brain. Given the high values of efficiency in the low spatial maps (SIMF3, SIMF4, and SIMF5), our results suggest that these maps can be considered as spatial global signal masks. The performance of the proposed method is compared with the SGSR method, and also with the results when GS is not removed. This is done by investigating the functional connections within an extracted peak voxel of the known network's regions and the selected seed region. While the correlation pattern identified by the other methods suffers from a low level of precision, our method demonstrates a high level of accuracy due to its data-driven adaptive nature.

## 2. Methods

### 2.1. fMRI Data Acquisition

The resting-state fMRI preprocessed data-set of 21 subjects from the NIH Human Connectome Project (HCP) (https://db.humanconnectome.org) (Essen et al., 2013) is used in this research. Each subject was involved in 4 runs of 15 min each using a 3 T Siemens, while their eyes were open and had a relaxed fixation on a projected bright cross-hair on a dark background. The data were acquired with 2.0 mm isotropic voxels for 72 slices, TR = 0.72 s, TE = 33.1 ms, 1,200 frames per run, 0.58 ms Echo spacing, and 2,290 Hz/Px Bandwidth (Moeller et al., 2010). Therefore, the fMRI data were acquired with a spatial resolution of 2 × 2 × 2 mm and a temporal resolution of 0.72 s, using multibands accelerated echo-planar imaging to generate a high quality and the most robust fMRI data (Moeller et al., 2010). The fMRI data were preprocessed to remove spatial artifacts produced by head motion, B0 distortions, and gradient non-linearities (Jovicich et al., 2006). As comparison of fMRI images across subjects and studies is possible when the images have been transformed from the subject's native volume space to the MNI space (Evans et al., 1993; Ashburner and Friston, 1999), fMRI images were wrapped and aligned into the MNI space with FSL's FLIRT 12 DOF affine and then a FNIRT non-linear registration (Jenkinson and Smith, 2001; Jenkinson et al., 2002; Jahanshad et al., 2013). In this study, the MNI-152-2 mm atlas (Mazziotta et al., 1995, 2001a,b) was utilized for fMRI data registration.

### 2.2. Estimation of the Temporal IMFs (TIMFs)

EMD is an adaptive data-driven signal processing method, which does not need any prior functional basis such as the wavelet transform (Mandic et al., 2008). The basic functions are derived adaptively from the data by the EMD sifting procedure. The EMD method developed and established by Huang et al. (1998) decomposes non-linear and non-stationary time series into their fundamental oscillatory components, called Intrinsic Mode Functions (IMFs). There are two criteria defining an IMF during the sifting process: 1) the number of extrema and zero crossings must be either equal or differ at most by one, and, 2) at any instant in time, the mean value of the envelope defined by the local maximum and the envelope of the local minimum is zero. The EMD algorithm for estimating the IMFs of the time series *x*(*t*) is:

1. *r*_0_(*t*) = *x*(*t*), *j* = 1.
2. For extracting the *j*-th IMF:
  (a) *h*_0_(*t*) = *r*_*j*_(*t*), *k* = 1,
  (b) Locate local maximum and minimum of *h*_*k*−1_(*t*),
  (c) Identify the average envelope using cubic spline interpolation to define upper and lower envelope of *h*_*k*−1_(*t*),
  (d) Calculate the mean value *m*_*k*−1_(*t*),
  (e) Put *h*_*k*_(*t*) = *h*_*k*−1_(*t*) − *m*_*k*−1_(*t*),
  (f) Check the stopping criteria. The stopping criteria determines the number of sifting steps to decompose an IMF Huang et al. (1998). If stopping criteria is satisfied then *h*_*j*_(*t*) = *h*_*k*_(*t*) otherwise, go to (a) to extract next IMF with *k* = *k* + 1.
3. *r*_*j*_(*t*) = *r*_*j*−1_(*t*) − *h*_*j*_(*t*).
4. If at least two extrema were in the *r*_*j*_(*t*), the next IMF is extracted otherwise the EMD algorithm is finished and *r*_*j*_(*t*) is the residue of *x*(*t*). Accordingly, *x*(*t*) is defined as:
  (1) $$\begin{array}{l} x\left(t\right)=\overset{n}{\underset{j=1}{\sum }}h_{j}\left(t\right)+r_{n}\left(t\right), \end{array}$$

where *h*_*j*_(*t*) is the *j*-th IMF, *n* is the number of IMFs, and *r*_*n*_(*t*) is the residue of the signal. Thus, the EMD method adaptively decomposes a time series into a set of IMFs and a residue where the first IMF (IMF1) corresponds to the fastest oscillatory mode and the last IMF (IMFn) to the slowest one, the sum of these components yields the original signal (Huang et al., 1998; Hassan and John, 2005). However, frequent occurrences of the mode-mixing phenomenon in analyzing real signals using EMD algorithm is problematic. To address this problem and improve the spectral separation of modes, the ensemble empirical mode decomposition (EEMD) method was proposed (Wu and Huang, 2009). This method extracts modes by performing the decomposition over an ensemble of noisy copies of the original signal combined with white Gaussian noises, and taking the average of all IMFs in the ensemble (Colominas et al., 2014).

The EEMD method solves the mode mixing problem, but certain issues remain. First, the number of IMFs extracted from each of the noisy signal copies is different, and this creates a problem when averaging the IMFs. The second problem is a reconstruction error in the EEMD method (Wu and Huang, 2009; Colominas et al., 2014). To fix this error the complementary EEMD (CEEMD) was proposed (Yeh et al., 2010). In the CEEMD algorithm, pairs of positive and negative white noise processes are added to the original signal to make two sets of ensemble IMFs. Accordingly, the CEEMD effectively eliminates residual noise in the IMFs which alleviate the reconstruction problem. Nonetheless, the problem of the different number of modes when averaging still persists. To overcome this problem, the CEEMD with adaptive noise (CEEMDAN) was developed (Torres et al., 2011; Colominas et al., 2014). In this approach, the first mode is computed exactly as in EEMD. Then, for the next modes, IMFs are computed by estimating the local means of the residual signal plus different modes extracted from the white noise realizations. CEEMDAN decomposition can create some spurious modes with high-frequency and low-amplitude due to overlapping in the scales. Additionally, some residual noise is still present in the modes. As a consequence, the new optimization algorithm, Improved Complete Ensemble Empirical Mode Decomposition with Adaptive Noise (ICEEMDAN), was proposed (Colominas et al., 2014).

During the sifting process using ICEEMDAN method the local mean of realizations is estimated, instead of using the average of modes from the first step. This change in the algorithm reduces the amount of noise present in the final computed modes. To deal with the issue of creation of spurious modes in the final results, ICEEMDAN method proceeds differently than the EEMD and CEEMDAN methods. In ICEEMDAN, white noise is not added directly; instead EMD modes of white noise are added to the original signal and to the IMFs during the sifting process (Wu and Huang, 2009; Colominas et al., 2014). Furthermore, in this method as in CEEMDAN, a constant coefficient is added to the noise that makes the desired signal to noise ratio between the added noise and the residue to which the noise is added. This coefficient is computed based on the standard deviation of the residue at each step of the sifting process. Therefore, the IMFs computed with ICEEMDAN have less noise and more physical content than IMFs obtained with other methods (Colominas et al., 2014) (More detailed description of ICEEMDAN method can be found at Colominas et al., 2014). The high accuracy rate, reduction in the amount of noise contained in the modes, and the alleviation of mode mixing phenomenon qualify this method to effectively decompose biological signals. In this paper the ICEEMDAN method with 300 ensembles and a level of noise of 0.2 (Wu and Huang, 2009) is used to extract the Temporal Intrinsic Mode Functions (TIMFs) from the fMRI data.

### 2.3. Estimation of the Spatial IMFs (SIMFs)

A fast, time efficient, and effective method is essential for processing real images that have a large size. Previous EMD-based methods were limited to small size images as the extrema detection, interpolation at each iteration, and the large number of iterations make their processing time consuming and complicated (Bhuiyan et al., 2008; Riffi et al., 2013, 2014; He et al., 2017). Therefore, those methods were just applicable to reduced size images, which resulted in losing some information during their process. Fast and Adaptive Tridimensional (3D) EMD, abbreviated as FATEMD, is a recent extension of the EMD method to three dimensions (Riffi et al., 2014). The FATEMD method is able to estimate volume components called tridimensional Intrinsic Mode Functions (3D-IMFs) quickly and accurately by limiting the number of iterations per 3D-IMF to one, and changing the process of computing upper and lower envelopes, which reduce the computation time for each iteration (Bhuiyan et al., 2008; Riffi et al., 2014; He et al., 2017). In the FATEMD method, the steps of extracting 3D-IMFs are almost the same as the previous EMD based methods, except for the number of iterations and the estimations of the maximum and minimum envelopes. The steps for decomposing a volume *V*(*m, n, p*) with dimensions *m, n*, and *p* using the FATEMD approach are as follows (Bhuiyan et al., 2008; Riffi et al., 2014):

1. Set *i* = 1 and *R*_*i*_(*m, n, p*) = *V*(*m, n, p*).
2. Determine the local maximum and minimum values by browsing *R*_*i*_(*m, n, p*) using a 3D window (cube) with a size of 3 × 3 × 3 which results in an optimum extrema maps (Map_max_(*m, n, p*) and Map_min_(*m, n, p*)). These local maximum (or minimum) values are strictly higher (or lower) than all of their neighborhoods contained in the cube.
3. Calculate the size of the Max and the Min filters which will be used in making extrema envelopes and their smoothness. The maximum and minimum filters are made by computing the nearest Euclidean distances between the maximum (*d*_adj.max_) (minimum (*d*_adj.min_)) points. The cubic window width (*w*_en_) then is determined by using one of the following four formulae for both maximum and minimum filters. Here, we used the 4-th formula as outlined below, although using the other formulas will result in approximately the same decomposition result:
  (2) $$\begin{array}{l} w_{\text{en}}=\min\left\{\min\left\{d_{\text{adj}.\max}\right\},\min\left\{d_{\text{adj}.\min}\right\}\right\},\\ w_{\text{en}}=\min\left\{\max\left\{d_{\text{adj}.\max}\right\},\max\left\{d_{\text{adj}.\min}\right\}\right\},\\ w_{\text{en}}=\max\left\{\min\left\{d_{\text{adj}.\max}\right\},\min\left\{d_{\text{adj}.\min}\right\}\right\},\\ w_{\text{en}}=\max\left\{\max\left\{d_{\text{adj}.\max}\right\},\max\left\{d_{\text{adj}.\min}\right\}\right\}. \end{array}$$
4. Create the envelopes of maxima and minima (Env_max_(*m, n, p*) and Env_min_(*m, n, p*)) of size (*w*_en_).
5. Use the mean filter to compute the smoothed envelopes:Env_max−*s*_(*m, n, p*) and Env_min−*s*_(*m, n, p*).
6. Calculate the mean filter by averaging the smoothed upper and lower envelopes (Env_*A*_(*m, n, p*)).
7. Calculate the *i-th* 3D-IMF: IMF_*i*_(*m, n, p*) = R_*i*_(*m, n, p*)−Env_*A*_(*m, n, p*).
8. Calculate R_*i*+1_(*m, n, p*) = R_*i*_(*m, n, p*)−IMF_*i*_(*m, n, p*).
9. If R_*i*+1_(*m, n, p*) contains more than two extrema then
             Go to the step 2 and set *i* = *i* + 1,
  Else
             The FATEMD decomposition is completed.

Therefore, FATEMD is an adaptive approach as all of the processes for computing filters and making the maximum, minimum, and the mean envelops are data driven. FATEMD decomposes a volume into a set of 3D-IMFs (Riffi et al., 2014). In general, a volume V can be reconstructed from the summation of the K 3D-IMFs and the residue as follows:

(3) $$\begin{array}{l} V\left(m,n,p\right)=\overset{\text{K}}{\underset{i=1}{\sum }}{\text{IMF}}_{i}\left(m,n,p\right)+{\text{R}}_{K+1}\left(m,n,p\right). \end{array}$$

*K* is the number of IMFs, and *R*(*m, n, p*) is the residue of the signal.

**Figure 1:**
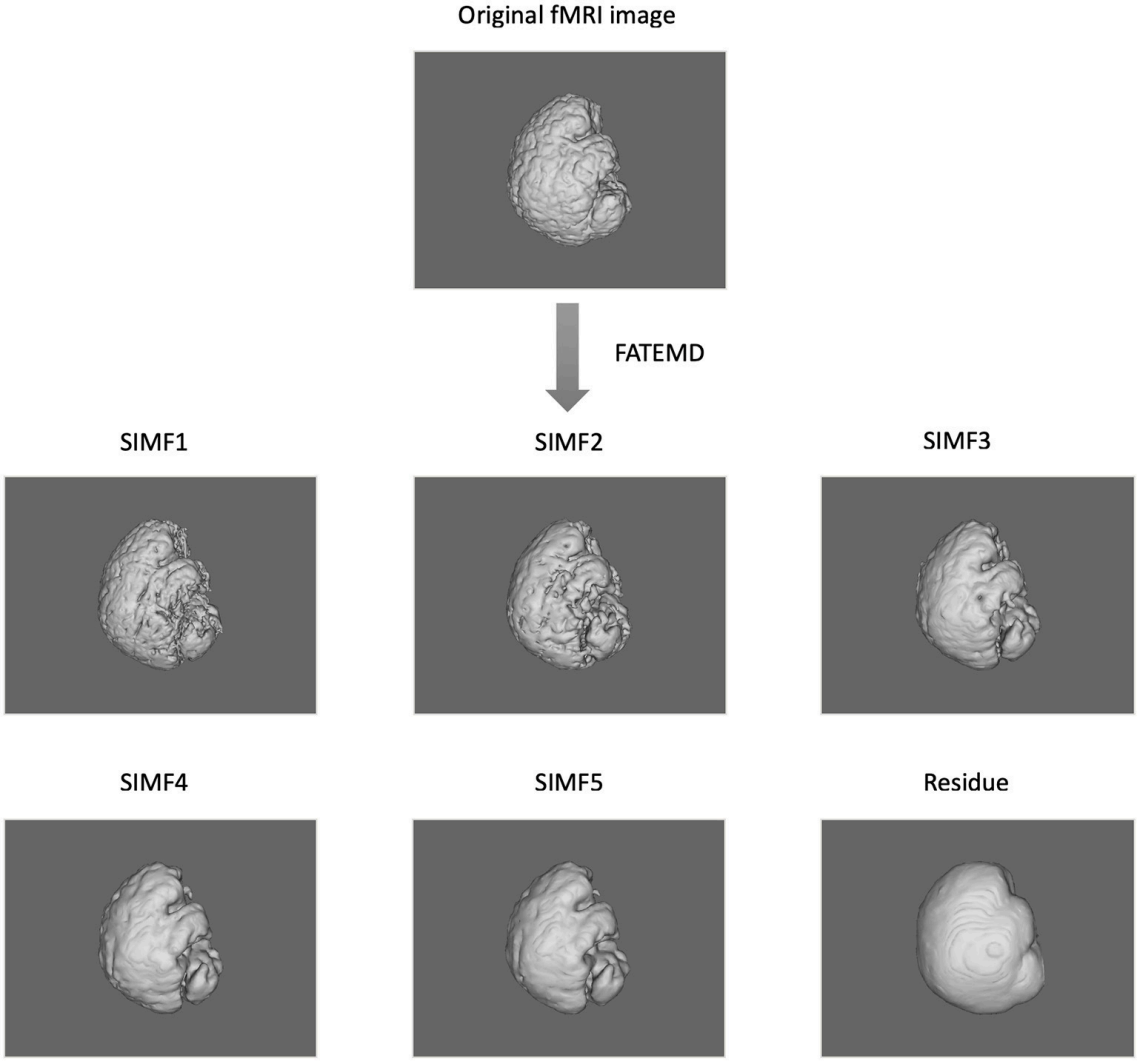
Spatial decomposition of a sample fMRI image using FATEMD method. The original fMRI image at one TR time is decomposed into 5 SIMFs (SIMF1 to SIMF5) and a residue.

In this paper, we apply the FATEMD method at each time instant to decompose the resting-state fMRI data into tridimensional IMFs called Spatial Intrinsic Mode Functions (SIMF). Figure 1 shows the spatial decomposition results of a sample resting-state fMRI image. The ICEEMDAN method is then utilized to decompose each SIMF into its corresponding TIMFs.

### 2.4. Spatiotemporal Pattern Analysis of the fMRI Data

To define an adaptive and voxel-specific GS, the spectral information of fMRI data is investigated by constructing the functional connectivity matrices using extracted TIMFs and SIMFs data. To fulfill this aim, first, the SIMFs of the fMRI data at each TR time are computed by applying the FATEMD method, then, all spatial components are merged together in time to construct the time series of each SIMF. Second, the peak voxel at each region, that is, the voxel of maximal activation, is selected by computing the Root Mean Square (RMS) for each voxel's signal over all time. It has been shown that peak voxel provides the best effect of any voxel in the ROI (Sharot et al., 2005). Additionally, the peak voxel activity correlates better with evoked scalp electrical potentials than approaches that average activity across the ROI. This means that the peak voxel represents the ROI's activity better than other choices (Arthurs and J Boniface, 2003). The peak voxel in each region is determined using previously published Talairach coordinates (after conversion to MNI coordinates and using AAL 116 atlas) (Fox et al., 2005). After determining the peak voxels of each region, the ICEEMDAN method is applied to its time series to compute the TIMFs. Thus, the TIMFs of all regions for each SIMF are computed.

**Figure 2:**
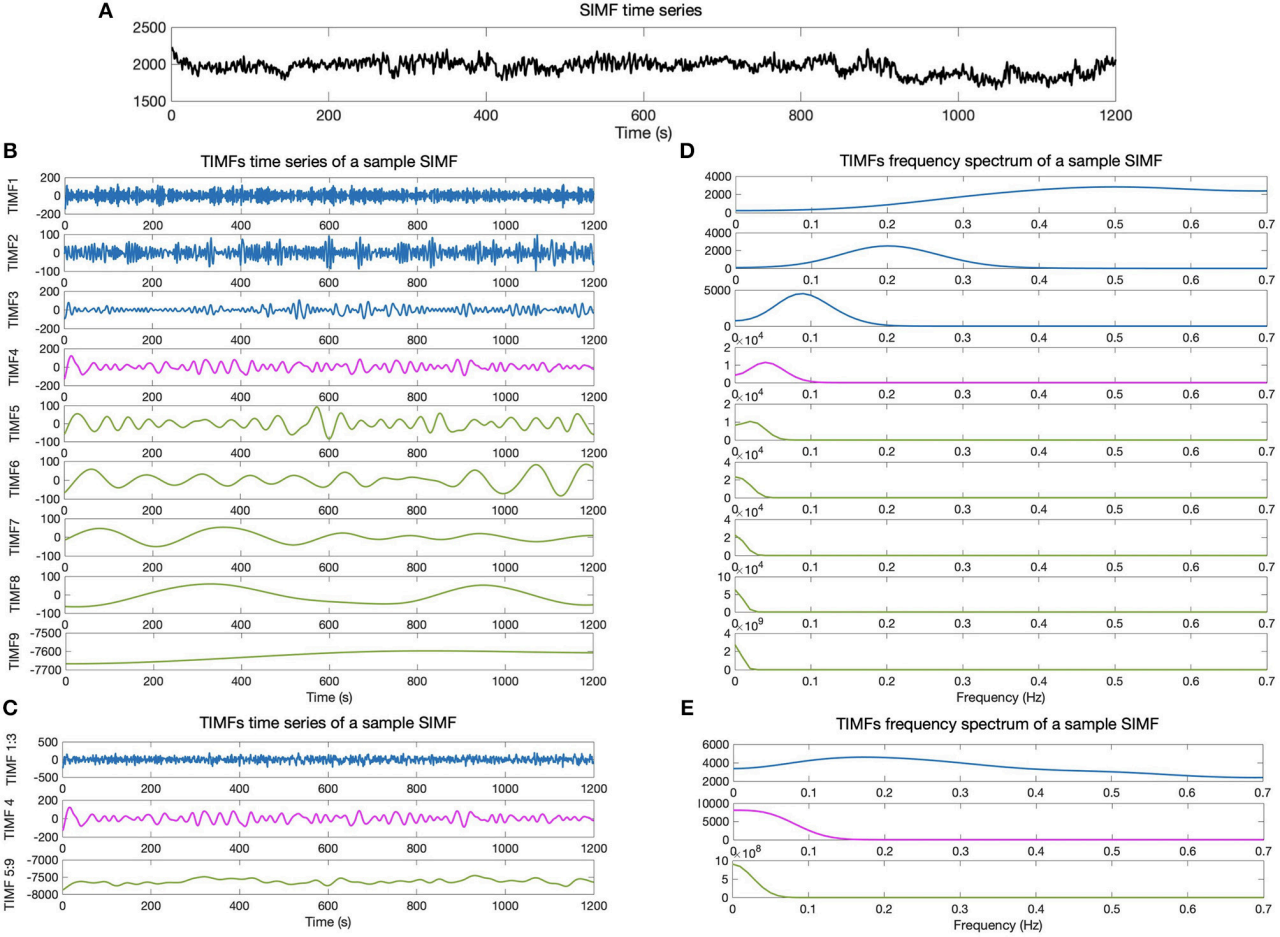
Temporal IMFs and their corresponding frequency spectrum of the sample SIMF time series. **(A)** Sample of SIMF time series before temporal decomposition **(B)** 9 decomposed TIMFs of a sample SIMF by applying the ICEEMDAN method with 300 ensembles and a level of noise of 0.2. **(C)** The 9 decomposed TIMFs are divided into three different frequency bands. According to slow1 to slow3 and slow5 frequency bands defined in the literature, TIMFs1 to 3 and 5 to 9 are combined, respectively. **(D)** Represents the frequency spectrum of the 9 TIMFs. **(E)** The frequency spectrum of TIMFs in part **(C)** that correspond to frequency bands used in the literature for slow1 to slow5 Penttonen and Buzsáki (2003), Zhan et al. (2014). TIMF, Temporal Intrinsic Mode Function; ICEEMDAN, Improved Complete Ensemble Empirical Mode Decomposition with Adaptive Noise.

**Figure 3:**
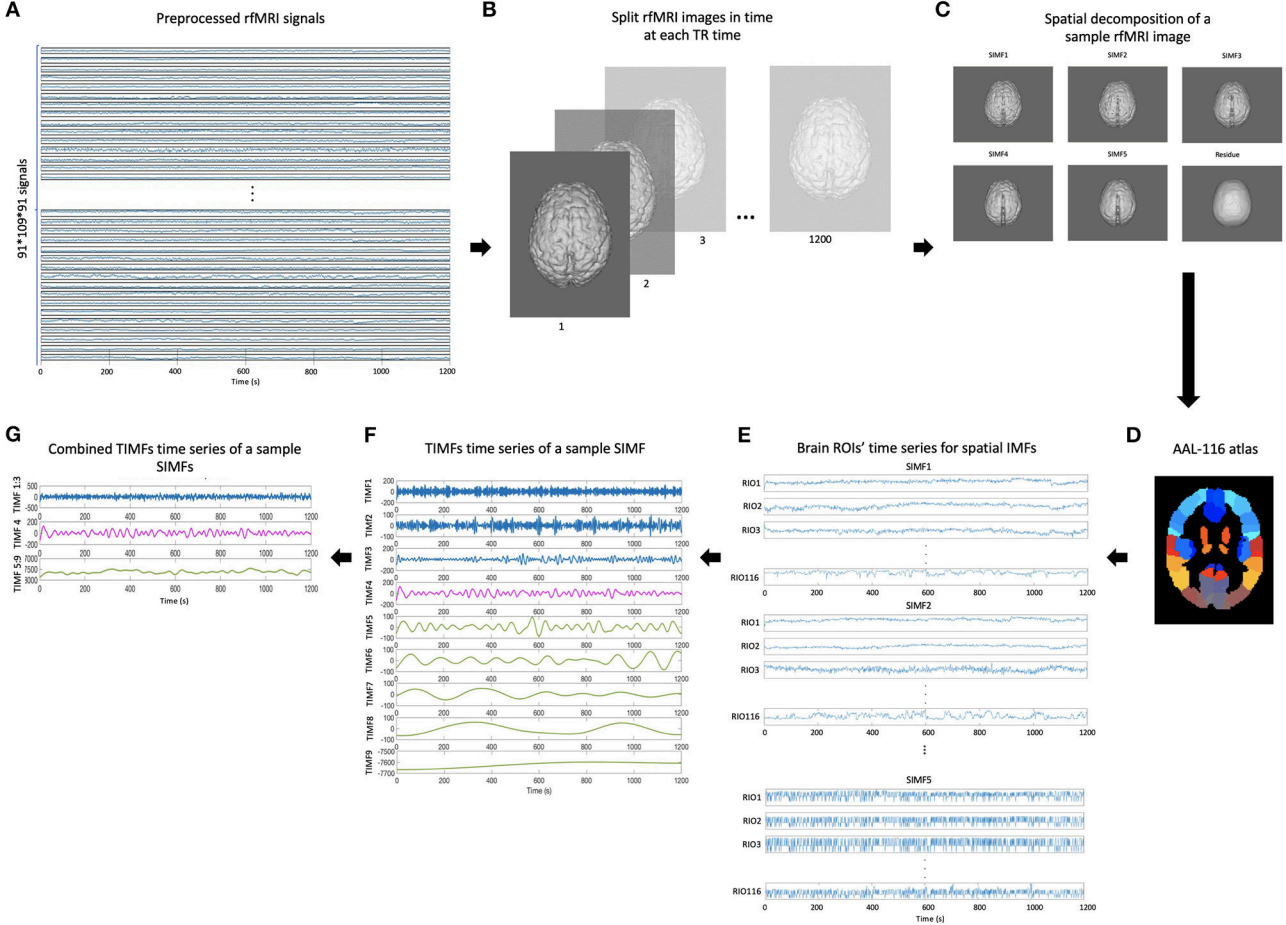
Pipeline for computing spatial and temporal IMFs (SIMF and TIMF) of the fMRI data. **(A)** A sample of fMRI data. **(B)** Splitting each fMRI data in time at each TR time. **(C)** SIMFs at each TR time which are computed by applying FATEMD approach. **(D)** Shows the AAL 116 atlas used after merging SIMFs in time to select the peak voxel of each region. **(E)** Time series of all brain ROIs for each SIMF. **(F)** The TIMFs' time series of a sample SIMF for one ROI computed by using ICEEMDAN approach. **(G)** Summation of time series of the TIMFs in **(F)** based on frequency bands of slow1 to slow5 defined in the literature. The summation of the TIMF1 to TIMF3, TIMF4, and the combination of TIMF5 to TIMF9 are labeled as TIMF1, TIMF2, and TIMF3 in the rest of the paper, respectively. rfMRI, resting-state fMRI; TIMF, Temporal Intrinsic Mode Function; SIMF, Spatial Intrinsic Mode Function; ICEEMDAN, Improved Complete Ensemble Empirical Mode Decomposition with Adaptive Noise; FATEMD, Fast and Adaptive Empirical Mode Decomposition; ROI, Region of Interest.

We then compare the predefined distinct frequency bands presented in fMRI studies (slow5 [0.01–0.027 Hz], slow4 [0.027–0.073 Hz], slow3 [0.073–0.198 Hz], slow2 [0.198–0.25 Hz], and slow1 [0.5–0.75 Hz]) (Penttonen and Buzsáki, 2003; Zhan et al., 2014), to the frequency content of the extracted TIMFs. In all subjects, TIMFs consistently corresponded to the same frequency bands. As seen in the Figure 2, the frequency range comprised in TIMF1 to TIMF3 is approximately the same as the frequency range of the sum of slow1 to slow3. The frequency range of TIMF4 is the same as slow4, and the frequency range of the sum of TIMF5 to TIMF9 has the same frequency range as the slow5 frequency band. Accordingly, we label the summation of TIMF1 to TIMF3 as TIMF1, TIMF4 as TIMF2, and the summation of TIMF5 to TIMF9 as TIMF3. Figure 3 represents the pipeline used in computing SIMFs and TIMFs for each resting-state fMRI data. Accordingly, the functional connectivity matrices are constructed by computing the average of correlation coefficients between all possible pairs of TIMFs correspond to different Spatial domains for all brain regions comprised in the AAL 116 atlas over all 21 subjects. Consequently, instead of the classical functional connectivity matrix, the decomposition presented here produces 5 × 3 = 15 connectivity matrices (each with size 116 × 116), 3 temporal domains and 5 spatial domains, encompassing the rich spatiotemporal dynamics of brain activity.

### 2.5. Topological Properties of the Brain Network

The GS is a synchronous fluctuation shared among all brain regions. Consequently, the GS component in the brain connectivity matrix should present a high integration value, where integration is the topological property of a network that describes how information from distributed brain regions is combined (Fair et al., 2007; Rubinov and Sporns, 2009; Cohen and D'Esposito, 2016). To compute the integration of the brain network at different spatiotemporal scales we use the global efficiency measure (Fair et al., 2007; Rubinov and Sporns, 2009). The global efficiency is computed as the average inverse shortest path length between all the node pairs of the network that is normalized by the maximal number of network's links. Therefore, the weighted global efficiency is computed via the following equation:

(4) $$E^{\text{w}}=\frac{1}{\text{N}(\text{N}-1)}\sum_{j=1}^{\text{N}}\sum_{i=1,i\neq j}^{\text{N}}{\left({d_{ij}}^{\text{w}}\right)}^{-1},$$

where N is the number of nodes in the network and *d*_*ij*_ is the minimum path length between nodes *i* and *j* (Fair et al., 2007; Rubinov and Sporns, 2009). The shortest path length is computed by counting the smallest number of edges needed to get from node *i* to node *j* which is inversely related to node weight. The information needed to estimate the weight of all pairs of brain regions are provided by functional connectivity matrices (Rubinov and Sporns, 2009; Cohen and D'Esposito, 2016), strong association between regions has a large weight which leads to a shorter length. When two nodes are disconnected the length of that path would be infinite and correspondingly, the efficiency would be zero (Fair et al., 2007; Rubinov and Sporns, 2009).

## 3. Results

### 3.1. Defining Adaptive Global Signal (AGS)

**Figure 4:**
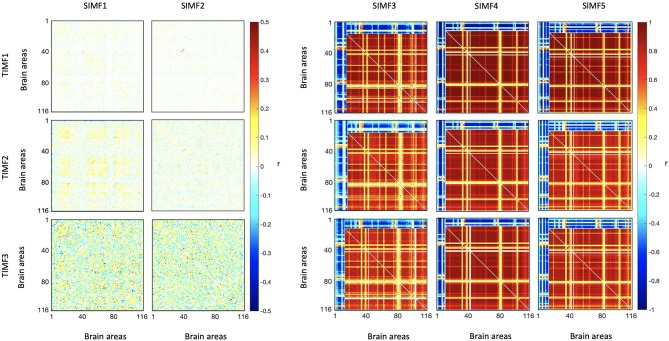
Functional connectivity matrices of the whole brain regions using AAL 116 atlas for different spatial and temporal IMFs. Pearson's correlation coefficient (*r*) with *P* ≤ 0.01 is computed between all the brain regions' spatiotemporal domains extracted from fMRI data. Spatial domains are extracted by applying FATEMD method on fMRI signal. The three temporal domains including TIMF1, TIMF2, and TIMF3 are computed by applying ICEEMDAN on each SIMF. SIMF, Spatial Intrinsic Mode Function; TIMF, Temporal Intrinsic Mode Function; ICEEMDAN, Improved Complete Ensemble Empirical Mode Decomposition with Adaptive Noise; FATEMD, Fast and Adaptive Empirical Mode Decomposition.

**Figure 5:**
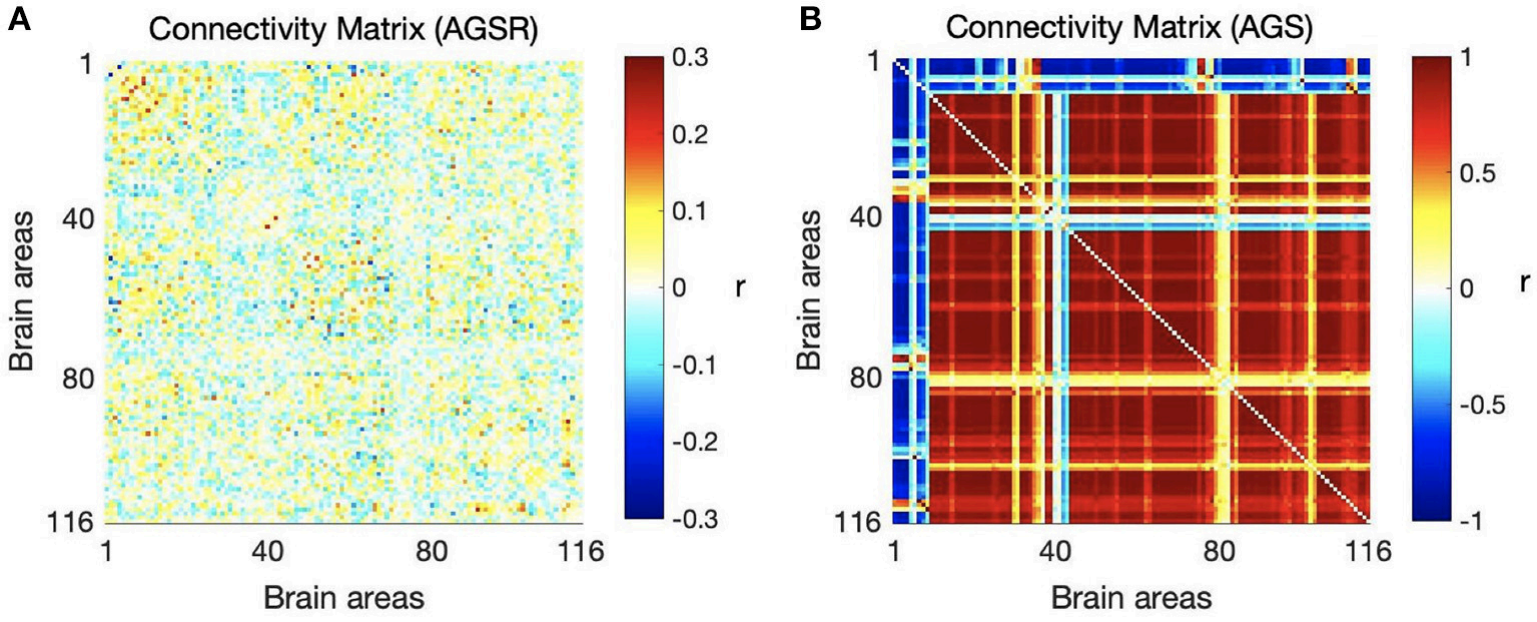
Average functional connectivity matrices of the whole brain regions using AAL 116 atlas over all subjects. **(A)** Average connectivity matrix of fMRI data applying AGSR which means the connectivity matrix of combination of SIMF1 and SIMF2 including all TIMFs of the fMRI data, **(B)** connectivity matrix of the AGS which is the combination of SIMF3 to SIMF5 including all TIMFs. AGSR, Adaptive Global Signal regression; AGS, Adaptive Global Signal; SIMF, Spatial Intrinsic Mode Function; TIMF, Temporal Intrinsic Mode Function.

We computed the functional connectivity matrices between all pairs of brain regions for different spatiotemporal domains extracted from fMRI data for each subject. Figure 4 shows the average connectivity matrices computed by Pearson's coefficient over the 21 subjects. As seen in the figures, SIMF1 and SIMF2 in all TIMFs showed low connectivity whereas SIMF3 to SIMF5 in all TIMFs showed high connectivity. Besides, they indicate that the magnitude of the correlation does not significantly depend on the TIMFs. Thus, based on the connectivity strength for different spatiotemporal domains, the summation of the SIMF1 to SIMF2 and the SIMF3 to SIMF5 including all TIMFs, were considered as two separate signals. We also averaged the six connectivity matrices resulting from the summation of TIMF1 to TIMF3 with SIMF1 and SIMF2 (Figure 4) and labeled it as AGSR (Figure 5A), and the nine connectivity matrices resulting when combining TIMF1 to TIMF3 with SIMF3 to SIMF5, which we labeled as AGS (Figure 5B).

**Figure 6:**
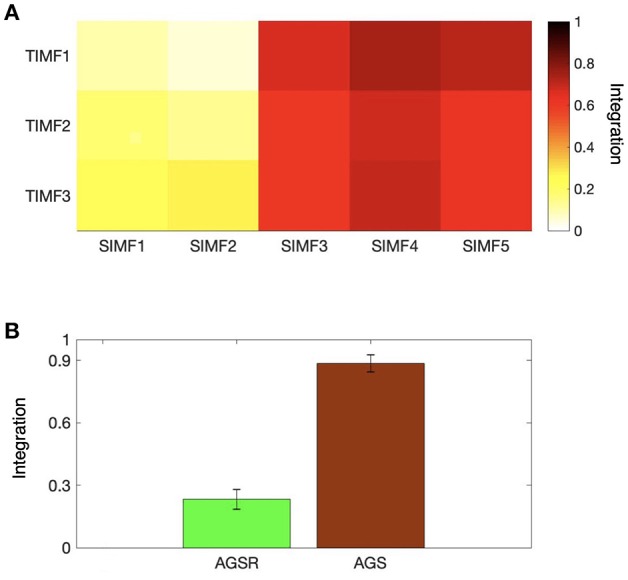
Integration of the brain network at different spatiotemporal scales. **(A)** Average efficiency of the whole brain network for different spatial and temporal IMFs defined in functional connectivity. **(B)** Comparing the magnitude of average efficiency of the brain network over all subjects when the AGS is removed from the fMRI time series (the sum of SIMF3 to SIMF5 in all TIMFS are removed and the sum of SIMF1 to SIMF2 including all TIMFs of the fMRI signal are considered to compute the connectivity), and the average efficiency of the AGS (summing up SIMF3 to SIMF5 in all TIMFs). High efficiency values in the SIMF3 to SIMF5 which represent the AGS in the fMRI data are seen in the figures. GS, Global Signal; AGS, Adaptive GS.

We also computed the global efficiency (Figure 6A) for different spatial and temporal IMFs using Equation (4) and also based on functional connectivity results. Figure 6A shows that there are high values of efficiency in the low frequencies of spatial domains, SIMF3, SIMF4, and SIMF5, which indicate active shared connections between all the nodes in the brain, suggesting the existence of GS in the low-frequency spatial domains, called Adaptive Global Signal(AGS). Furthermore, SIMF3 to SIMF5 with high temporal frequency mode (TIMF1) which is included in the AGS can be considered as an adaptive filter to reduce the effects of the highly integrated physiological noises in high frequency modes instead of applying low-pass filtering (Shmueli et al., 2007; Boubela et al., 2013; Liu et al., 2017).

As seen in Figure 6B and Table 1, the high values of integration of AGS (summation of SIMF3 to SIMF5 including all TIMFs) confirm that they can be considered as a GS which has to be removed from the fMRI data to have more accurate brain connectivity results. In the last results' section (represented in Figures 8, 9) we show that, including low frequency spatial domains may cause spurious connectivity results between brain regions.

**Table 1:** Integration of AGSR and AGS. The average efficiency of the brain network over all subjects, when the AGSR are performed, and the average efficiency of the AGS. AGS, Adaptive Global Signal; AGSR, AGS Regression.

**Network measure**	**Label**	**Interpretation**	**Value**
Efficiency	AGSR	The brain network's average efficiency when AGSR is performed	0.2325±0.0480
Efficiency	AGS	The brain network's average efficiency of the AGS	0.8850±0.0417

### 3.2. Regressing Out the AGS and SGS From fMRI Data

**Figure 7:**
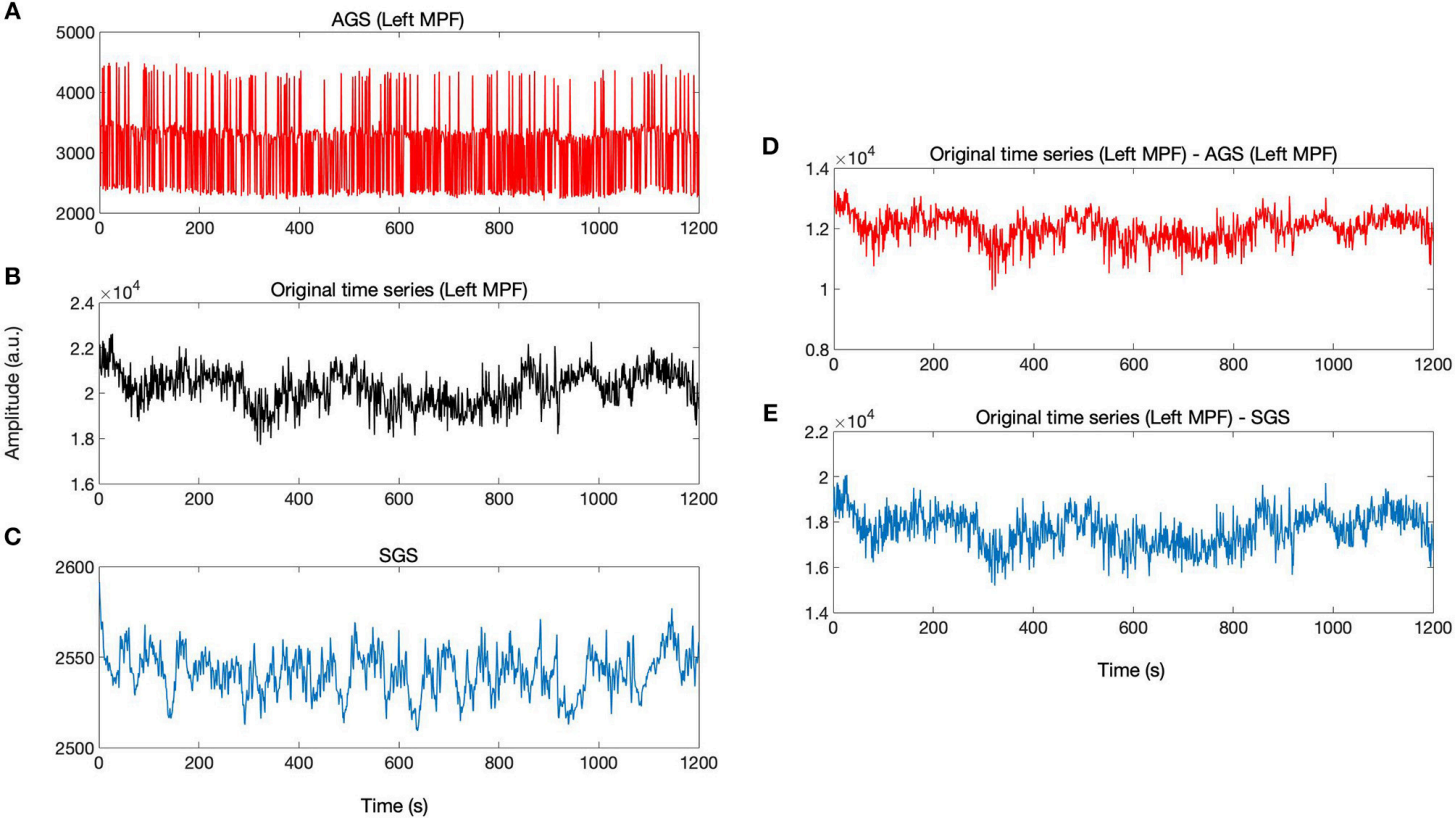
AGSR and SGSR of a sample fMRI data. **(A)** The voxel-specific AGS of Medial Prefrontal (MPF) cortex region and **(B)** the original fMRI time series of the peak voxel in MPF cortex region. **(C)** The SGS which is common for all region's voxels. **(D,E)** Show the time series with the SGSR and AGSR, respectively. These time series are computed by subtracting the AGS and SGS from the original time series. MPF, Medial Prefrontal cortex; AGS, Adaptive Global Signal; SGS, Static(conventional) Global Signal; AGSR, AGS Regression; SGSR, SGS Regression.

According to the definition of AGS, for each brain voxel signal, there is a corresponding AGS while the SGS is common for the whole brain voxels. The AGS for each voxel is computed by summing up the SIMF3, SIMF4, and SIMF5 with all TIMFs while the SGS is computed by taking the average of all brain voxels' time series. It should be noted that in computing AGS, the residues of spatiotemporal decomposition of the fMRI data are added to the last TIMF and SIMF. The three time courses in Figures 7A–C correspond to the AGS, the fMRI sample time course [the peak voxel's time course in Medial Prefrontal cortex (MPF) ROI], and the conventional or Static GS (SGS), respectively. Figures 7D,E show resting-state fluctuations of the sample fMRI time series from MPF ROI after regressing out (subtracting) the AGS and the SGS. It also has to be mentioned that to be consistent with the previous fMRI studies, data are conventionally low-pass filtered except when the AGSR method is applied.

### 3.3. Connectivity Map of Task-Positive and Task-Negative Networks

The default mode network or Task Negative Network (TNN) is a state of brain activation whereby the individual is not attending to any external cues in the environment but certain brain regions are still activated and they are less active during task performance rather than during the resting-state. It has been shown that (Fox et al., 2005) the default mode network responses are significantly activated in three of the seeded regions: the Posterior Cingulate Cortex (PCC), Medial Prefrontal cortex (MPF), and Lateral Parietal cortex (LP). The efficacy of our approach is examined by computing the connectivity map. In computing functional connectivity maps, we computed Pearson's correlation which is popular in fMRI studies and also allows our findings to be comparable with other papers to test the validity of the proposed method. We computed the average connectivity between the time course of the PCC region as a seed region and the main regions of the Task Positive Network (TPN) which are the Middle Temporal (MT), right Frontal Eye Field (FEF), left Intraparietal Sulcus (IPS), Supplementary Motor Area (SMA), Inferior Parietal Lobule (IPL), Visual regions, and the left Auditory region and the TNN ROIs which are MPF, PCC, and left LP which includes the Angular Gyrus, Hippocampus, and Cerebellar tonsils ROIs (Fox et al., 2009; Erdoğan et al., 2016).

**Figure 8:**
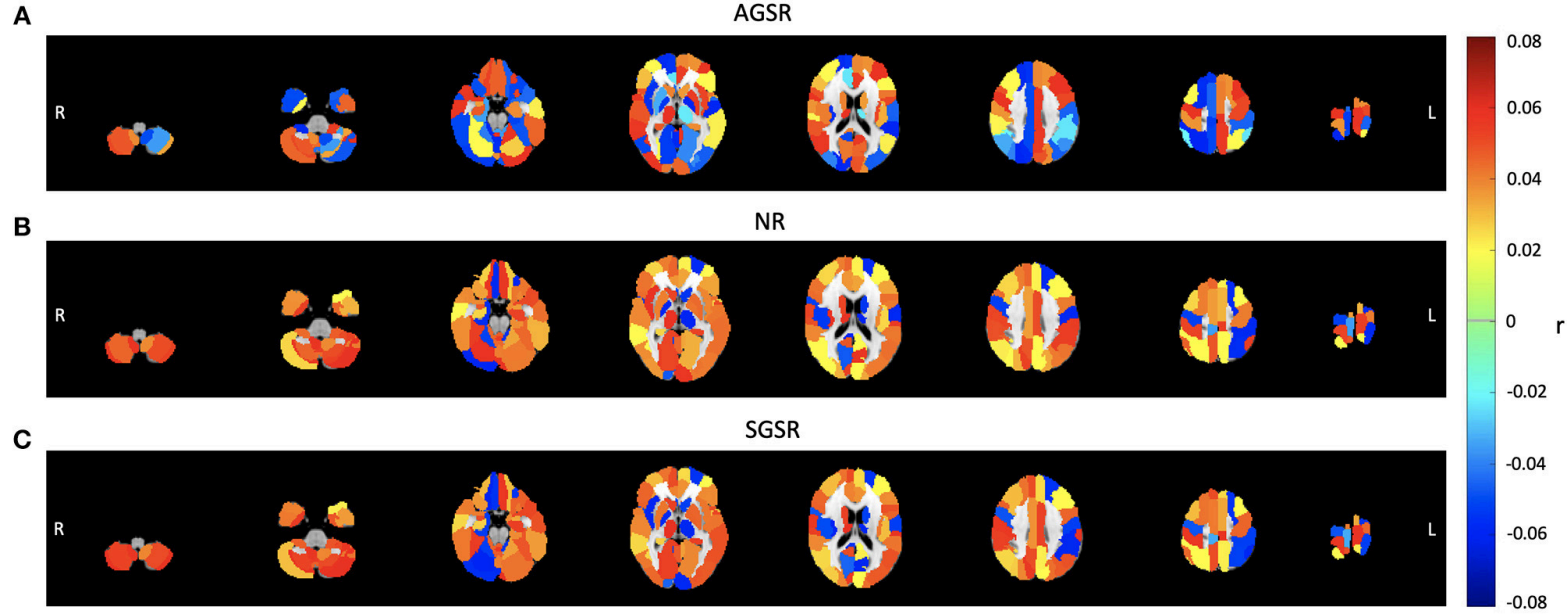
Comparing the average functional connectivity between the PCC ROI as a seed region and the brain ROIs using the AAL 116 atlas for fMRI data of all subjects. The average functional connectivity applying **(A)** AGSR, **(B)** NR, and **(C)** SGSR. Slices shown in the maps are at Z = 09, 15, 25, 35, 45, 55, 65, 75, respectively. AGSR, Adaptive Global Signal Regression; NR, No Regression; SGSR, Static (conventional) Global signal regression.

Considering the AGS definition, the combination of the SIMF1 and SIMF2 was used to compute the functional connectivity between PCC and TNN and TPN including visual ROIs by using Pearson's correlation coefficient (r), *P* ≤ 0.01. Figure 8 is functional connectivity brain map for different brain layers along the Z axis which show the mean connectivity over all subjects between brain regions and the PCC ROI as a seed region when the AGSR, NR, and the SGSR are performed.

**Figure 9:**
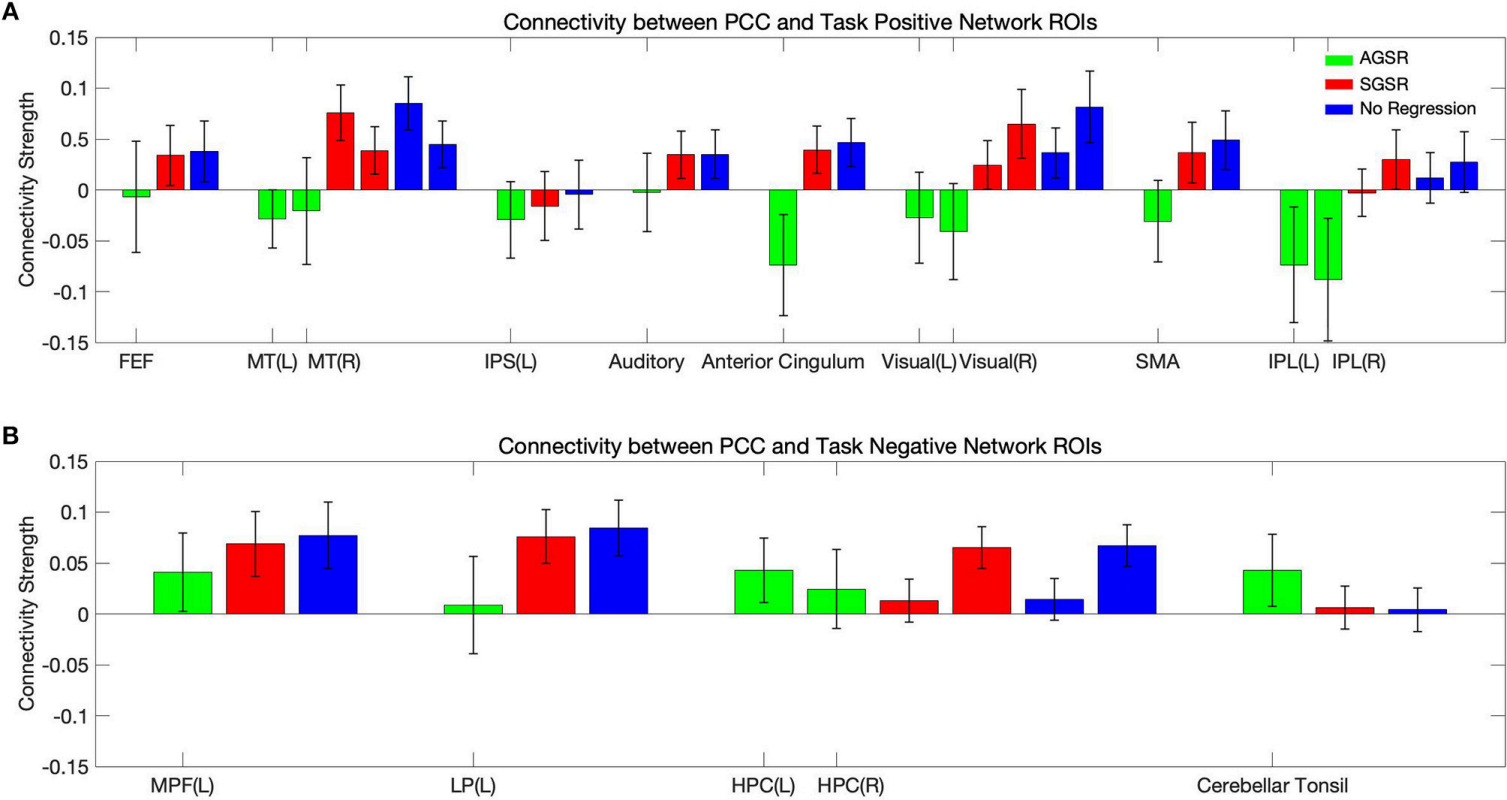
The average connectivity map between the PCC as a seed region and **(A)** the TPN and **(B)** TNN ROIs for fMRI data of all subjects. Connectivity results of applying AGSR and SGSR are shown in green and red, respectively, and the blue ones are the results of computing connectivity without applying any GSR (NR). Connectivity map is made by computing Pearson's correlation coefficient (r) with *P* ≤ 0.01 between the PCC region as a seed region and the main regions of the TPN and TNN. PCC, Posterior Cingulate Cortex; MPF, Medial Prefrontal cortex; LP, Lateral Parietal cortex; MT, Middle Temporal; FEF, Frontal Eye Field; IPS, Intraparietal Sulcus; SMA, Supplementary Motor Area; IPL, Inferior Parietal Lobule; ROI, Region Of Interest; NR, No Regression; AGS, Adaptive Global Signal Regression; SGS, Static (conventional) Global Signal Regression.

Figure 9 shows expected average connectivity between the PCC ROI and different regions of the TPN and the TNN (positive correlation between the PCC and the TNN and negative correlation between the PCC and TPN) applying the new approach of GSR in resting-state fMRI data.

While the NR and SGSR (conventional GSR which is based on averaging) are unable to identify the expected connectivity in some regions for TPN and TNN ROIs, the AGSR approach obtains expected functional connectivity for all regions in TNN and TPN which confirms the effectiveness of the proposed method for GSR (Figure 9). As AGSR is an adaptive and voxel-specific method, we have a unique local signal for each voxel which by being removed from fMRI data augments the precision of the rsfc-MRI results.

## 4. Discussion

In contrast to previous works (Zarahn et al., 1997; Fox et al., 2009; Liu et al., 2017), the present study provides a new method for GSR, called AGSR, that works voxel-specifically and adaptively. It is believed that fMRI data are a superposition of the GS and network-specific fluctuations. However, the main reason for the controversy over the use of GSR in fMRI studies is that the average-based GS is a mixture of signals from multiple brain regions without considering the possibility of spatial heterogeneity in the GS (Fox et al., 2009; Murphy et al., 2009; Weissenbacher et al., 2009; Saad et al., 2012; Murphy and Fox, 2017). It has been shown that regressing out average-based GS results in negative correlations that do not have a biological basis and are artifacts in the voxels' time series which lead to distortion in the connectivity results or activation measures (Fox et al., 2009; Murphy et al., 2009; Murphy and Fox, 2017). In this paper, we showed that the AGSR method works voxel-specifically and can compute the neuronal correlations of the brain's networks more accurately. This is because using the FATEMD method in computing AGS maximizes the spatial contributions to the GS. In other words, decomposing fMRI data in space using the FATEMD approach, which is done by considering features of each voxel's neighbors, makes the computed AGS sensitive to brain regions' heterogeneity.

When assessing the efficiency for different spatiotemporal domains of the fMRI data, no large differences in different temporal IMFs at the same spatial IMF were obtained. Thus, we concluded that the variability of efficiency is just related to the spatial frequency domains. The high values of the efficiency in the low spatial frequencies demonstrated the existence of the GS. On the other hand, high spatial frequencies, SIMF1 and SIMF2, represented the most network-specific data. Accordingly, the low spatial frequencies, SIMF3 to SIMF5 including all TIMFs, were considered as the AGS.

Additionally, it has been shown that motion, cardiac, and respiratory noise components which have high frequency cycles and are spatially coherent, cause spatially widespread fluctuations in the BOLD signals that contribute to the global signal (Shmueli et al., 2007; Liu et al., 2017). Conventionally, filtering the high frequency components of the fMRI data to remove above mentioned physiological noises and the GSR are done separately as two preprocessing steps in fMRI studies (He and Liu, 2012; Caballero-Gaudes and Reynolds, 2017; Liu et al., 2017), however, common low-pass filtering methods through removing high frequency components cause missing a considerable amount of information on resting-state brain functional network (Tagliazucchi et al., 2011, 2012; Boubela et al., 2013; Turchi et al., 2018). In our proposed method, in addition to GSR, physiological noise components that are common across voxels and are mainly included in the high frequency modes are also removed from the data by removing the SIMF3 to SIMF5 of TIMF1 through AGSR. Thus, our proposed method, through AGSR, filters the highly connected part of high frequency modes adaptively without applying low-pass filter separately. It can help to provide more informative data by involving high frequency modes in the data.

We examined the efficacy of our method by computing the seed-based functional connectivity for the TPN and TNN regions. Our results in agreement with previous studies (Chang and Glover, 2009; Fox et al., 2009; Chai et al., 2012), show that the negative correlations are intrinsic to the brain and do not appear just as a result of the GSR. We found that the AGSR method identifies the connectivity between the TPN and TNN regions according with the expected results of prior studies (Fox et al., 2005, 2009). We compared the connectivity results of the AGSR with the SGSR and when there is NR in the fMRI data. Despite the connectivity results of the SGSR method and when there is NR, applying our proposed method resulted in an enhancement to the detection of network-specific fluctuations of the brain. Furthermore, although the strength of the correlations is related to cognitive function, in auditory regions, lower activity seen in the result of applying AGSR appears to be related to the better removal of the acoustic noise heard by subjects during fMRI. This shows that the acoustic noise of the fMRI device which is almost constant in all TR times and interferes with auditory system activity can be removed better through AGSR (Ravicz et al., 2000; Moelker and Pattynama, 2003). Thus, it is inferred from the results that AGSR method is able to remove physiological and remained systemic noises after preprocessing more correctly and without introducing artifactual correlations as confirmed by correlations between PCC and the reference regions.

In conclusion, AGS is a unique local signal for each voxel's BOLD signal. In the AGSR method, the first and second spatial IMFs of each fMRI data, decomposed by FATEMD method, are simply summed up to have a band-pass filtered fMRI data without GS. AGSR is a reliable method that works voxel-specifically for all subjects which leads to provide information about brain function with more accuracy. There are some limitations to the methods used in this study that should be noted. Although the FATEMD and ICEEMDAN are optimized approaches for finding the best IMF sets, they still need more improvement in the sifting procedure to yield better decomposition performance. For instance, finding the optimum values of added white noise and the ensemble number to overcome the mode mixing problem and speed up the calculation in ICEEMDAN approach are two drawbacks of this approach.

We computed the GS for each region of the AAL 116 atlas specifically, however, as this method has a “voxel-specific” nature, it can be applied to all voxels of the brain. Computing voxel-specific GS just needs more memory and computer power, such as a larger computer cluster but no additional changes to the underlying algorithm are needed. It is more feasible to compute the AGSR for all the voxels when we are interested in some specific regions of the brain and not the whole brain.

Therefore, the proposed method in this paper provides the opportunity to characterize the whole brain function and reflect the intrinsic property of the spatiotemporal nature of the fMRI data through removing the voxel-specific GS and not removing the whole high frequency modes. Future studies can be devoted to the application of our proposed method to the other image processing areas.

## Author Contributions

NM and RS designed the research. NM analyzed the data, interpreted the results and wrote the main manuscript. MD assisted with analysis and interpretation of data. RS supervised the development of the work, helped in data interpretation and manuscript evaluation.

### Conflict of Interest Statement

The authors declare that the research was conducted in the absence of any commercial or financial relationships that could be construed as a potential conflict of interest.

## References

[B1] Anderson J. S. Druzgal T. J. Lopez Larson M. Jeong E. K. Desai K. Yurgelun-Todd D. (2010). Network anticorrelations, global regression, and phase-shifted soft tissue correction. Hum. Brain Mapp. 32, 919–934. 10.1002/hbm.21079 20533557 PMC3220164

[B2] Arthurs O. J Boniface S. (2003). What aspect of the fmri bold signal best reflects the underlying electrophysiology in human somatosensory cortex? Clin. Neurophysiol. 114, 1203–1209. 10.1016/S1388-2457(03)00080-4 12842716

[B3] Ashburner J. Friston K. J. (1999). Nonlinear spatial normalization using basis functions. Hum. Brain Mapp. 7, 254–266. 10.1002/(SICI)1097-0193(1999)7:4<254::AID-HBM4>3.0.CO;2-G 10408769 PMC6873340

[B4] Bhuiyan S. M. A. Adhami R. R. Khan J. F. (2008). Fast and adaptive bidimensional empirical mode decomposition using order-statistics filter based envelope estimation. EURASIP J. Adv. Signal Process. 2008:728356 10.1155/2008/728356

[B5] Biswal B. Zerrin Yetkin F. Haughton V. M. Hyde J. S. (1995). Functional connectivity in the motor cortex of resting human brain using echo-planar MRI. Magn. Resonan. Med. 34, 537–541. 10.1002/mrm.1910340409 8524021

[B6] Boubela R. Kalcher K. Huf W. Kronnerwetter C. Filzmoser P. Moser E. (2013). Beyond noise: using temporal ica to extract meaningful information from high-frequency fMRI signal fluctuations during rest. Front. Hum. Neurosci. 7:168. 10.3389/fnhum.2013.00168 23641208 PMC3640215

[B7] Brooks J. Faull O. Pattinson K. Jenkinson M. (2013). Physiological noise in brainstem fMRI. Front. Hum. Neurosci. 7:623. 10.3389/fnhum.2013.00623 24109446 PMC3790256

[B8] Caballero-Gaudes C. Reynolds R. C. (2017). Methods for cleaning the bold fMRI signal. NeuroImage 154, 128–149. 10.1016/j.neuroimage.2016.12.018 27956209 PMC5466511

[B9] Chai X. J. Castañón A. N. Ongür D. Whitfield-Gabrieli S. (2012). Anticorrelations in resting state networks without global signal regression. NeuroImage 59, 1420–1428. 10.1016/j.neuroimage.2011.08.048 21889994 PMC3230748

[B10] Chang C. Glover G. H. (2009). Effects of model-based physiological noise correction on default mode network anti-correlations and correlations. NeuroImage 47, 1448–1459. 10.1016/j.neuroimage.2009.05.012 19446646 PMC2995588

[B11] Chang C. Leopold D. Schölvinck M. Mandelkow H. Picchioni D. Liu X.. (2016). Tracking brain arousal fluctuations with fmri. Proc. Natl. Acad. Sci. U.S.A. 113, 4518–4523. 10.1073/pnas.1520613113 27051064 PMC4843437

[B12] Cohen J. R. D'Esposito M. (2016). The segregation and integration of distinct brain networks and their relationship to cognition. J. Neurosci. 36, 1283–2094. 10.1523/JNEUROSCI.2965-15.2016 27903719 PMC5148214

[B13] Colominas M. Schlotthauer G. Torres M. E. (2014). Improved complete ensemble EMD: a suitable tool for biomedical signal processing. Biomed. Signal Process. Control 14, 19–29. 10.1016/j.bspc.2014.06.009

[B14] Comon P. Jutten C. (2010). Handbook of Blind Source Separation, Independent Component Analysis and Applications, 1st Edn. Boston, MA: Academic Press; Elsevier.

[B15] Cordes D. Zhuang X. Kaleem M. Sreenivasan K. Yang Z. Mishra V.. (2018). Advances in functional magnetic resonance imaging data analysis methods using empirical mode decomposition to investigate temporal changes in early parkinson's disease. Alzheimer's Dementia Transl. Res. Clin. Intervent. 4, 372–386. 10.1016/j.trci.2018.04.009 30175232 PMC6115608

[B16] Damoiseaux J. S. Rombouts S. A. R. B. Barkhof F. Scheltens P. Stam C. J. Smith S. M.. (2006). Consistent resting-state networks across healthy subjects. Proc. Natl. Acad. Sci. U.S.A. 103, 13848–13853. 10.1073/pnas.0601417103 16945915 PMC1564249

[B17] De Luca M. Beckmann C. De Stefano N. Matthews P. Smith S. (2006). fMRI resting state networks define distinct modes of long-distance interactions in the human brain. NeuroImage 29, 1359–1367. 10.1016/j.neuroimage.2005.08.035 16260155

[B18] Erdoğan S. B. Tong Y. Hocke L. M. Lindsey K. P. DeB Frederick B. (2016). Correcting for blood arrival time in global mean regression enhances functional connectivity analysis of resting state fMRI-BOLD signals. Front. Hum. Neurosci. 10:311. 10.3389/fnhum.2016.00311 27445751 PMC4923135

[B19] Essen D. C. V. Smith S. M. Barch D. M. Behrens T. E. Yacoub E. Ugurbil K. (2013). The WU-Minn human connectome project: an overview. NeuroImage 80, 62–79. 10.1016/j.neuroimage.2013.05.041 23684880 PMC3724347

[B20] Evans A. C. Collins D. L. Mills S. R. Brown E. D. Kelly R. L. Peters T. M. (1993). 3D statistical neuroanatomical models from 305 mri volumes, in 1993 IEEE Conference Record Nuclear Science Symposium and Medical Imaging Conference, Vol. 3, 1813–1817.

[B21] Fair D. A. Dosenbach N. U. F. Church J. A. Cohen A. L. Brahmbhatt S. Miezin F. M.. (2007). Development of distinct control networks through segregation and integration. Proc. Natl. Acad. Sci. U.S.A. 104, 13507–13512. 10.1073/pnas.0705843104 17679691 PMC1940033

[B22] Fox M. Snyder A. Zacks J. Raichle M. (2006). Coherent spontaneous activity accounts for trial-to-trial variability in human evoked brain responses. Nat. Neurosci. 9, 23–25. 10.1038/nn1616 16341210

[B23] Fox M. D. Raichle M. E. (2007). Spontaneous fluctuations in brain activity observed with functional magnetic resonance imaging. Nat. Rev. Neurosci. 8, 700–711. 10.1038/nrn2201 17704812

[B24] Fox M. D. Snyder A. Z. Vincent J. L. Corbetta M. Van Essen D. C. Raichle M. E. (2005). The human brain is intrinsically organized into dynamic, anticorrelated functional networks. Proc. Natl. Acad. Sci. U.S.A. 102, 9673–9678. 10.1073/pnas.0504136102 15976020 PMC1157105

[B25] Fox M. D. Zhang D. Snyder A. Z. Raichle M. E. (2009). The global signal and observed anticorrelated resting state brain networks. J. Neurophysiol. 101, 3270–3283. 10.1152/jn.90777.2008 19339462 PMC2694109

[B26] Friston K. J. (2011). Functional and effective connectivity: a review. Brain Connect. 1, 13–36. 10.1089/brain.2011.0008 22432952

[B27] Gallagher T. A. Nemeth A. J. Hacein-Bey L. (2008). An introduction to the fourier transform: relationship to MRI. Am. J. Roentgenol. 5, 1396–1405. 10.2214/AJR.07.2874 18430861

[B28] Hassan H. John W. P. (2005). Empirical mode decomposition (EMD) of potential field data: airborne gravity data as an example. Can. Soc. Explor. Geophys. 24, 704–706. 10.1190/1.2144422

[B29] He H. Liu T. T. (2012). A geometric view of global signal confounds in resting-state functional MRI. NeuroImage 59, 2339–2348. 10.1016/j.neuroimage.2011.09.018 21982929 PMC3254803

[B30] He Z. Li J. Liu L. Shen Y. (2017). Three-dimensional empirical mode decomposition (TEMD): a fast approach motivated by separable filters. Signal Process. 131, 307–319. 10.1016/j.sigpro.2016.08.024

[B31] Huang N. Shen Z. Long S. Wu M. Shih H. Zheng Q. (1998). The empirical mode decomposition and the hilbert spectrum for nonlinear and non-stationary time series analysis. Proc. R. Soc. Lond. A 454, 903–995. 10.1098/rspa.1998.0193

[B32] Jahanshad N. Kochunov P. V. Sprooten E. Mandl R. C. Nichols T. E. Almasy L.. (2013). Multi-site genetic analysis of diffusion images and voxelwise heritability analysis: a pilot project of the ENIGMA–DTI working group. NeuroImage 81, 455–469. 10.1016/j.neuroimage.2013.04.061 23629049 PMC3729717

[B33] Jenkinson M. Bannister P. Brady M. Smith S. (2002). Improved optimization for the robust and accurate linear registration and motion correction of brain images. NeuroImage 17, 825–841. 10.1006/nimg.2002.1132 12377157

[B34] Jenkinson M. Smith S. (2001). A global optimisation method for robust affine registration of brain images. Med. Image Anal. 5, 143–156. 10.1016/S1361-8415(01)00036-6 11516708

[B35] Jovicich J. Czanner S. Greve D. Haley E. van der Kouwe A. Gollub R.. (2006). Reliability in multi-site structural MRI studies: effects of gradient non-linearity correction on phantom and human data. NeuroImage 30, 436–443. 10.1016/j.neuroimage.2005.09.046 16300968

[B36] Kandel E. R. Schwartz J. H. Jessell T. M. (2000). Principles of Neural Science, Vol. 4, 4th Edn. McGraw-Hill Medical.

[B37] Lin S.-H. N. Lin G.-H. Tsai P.-J. Hsu A.-L. Lo M.-T. Yang A. C. Lin C.-P.. (2016). Sensitivity enhancement of task-evoked fMRI using ensemble empirical mode decomposition. J. Neurosci. Methods 258, 56–66. 10.1016/j.jneumeth.2015.10.009 26523767

[B38] Liu T. T. Nalci A. Falahpour M. (2017). The global signal in fMRI: nuisance or information? NeuroImage 150, 213–229. 10.1016/j.neuroimage.2017.02.036 28213118 PMC5406229

[B39] Liutkus A. Durrieu J.-L. Daudet L. Richard G. (2013). An overview of informed audio source separation, in Proceedings of the 14th International Workshop Image Analysis Multimedia Interaction Service (Paris), 1–4.

[B40] Macey P. Macey K. Kumar R. Harper R. (2004). A method for removal of global effects from fMRI time series. NeuroImage 22, 360–366. 10.1016/j.neuroimage.2003.12.042 15110027

[B41] Mandic D. Golz M. Kuh A. Obradovic D. Tanaka T. (2008). Signal Processing Techniques for Knowledge Extraction and Information Fusion, 1st Edn. New York, NY: Springer Publishing Company, Incorporated.

[B42] Mazziotta J. C. Toga A. Evans A. Fox P. Lancaster J. N. Zilles K. (2001a). A probabilistic atlas and reference system for the human brain: international consortium for brain mapping (ICBM). Philos. Trans. R. Soc. Lond. Ser. B Biol. Sci. 1412, 1293–1322. 10.1098/rstb.2001.0915 PMC1088516 11545704

[B43] Mazziotta J. C. Toga A. Evans A. Fox P. Lancaster J. N. Zilles K. (2001b). Four-dimensional probabilistic atlas of the human brain: international consortium for brain mapping (ICBM). J. Am. Med. Inform. Assoc. 8, 401–430. 10.1136/jamia.2001.0080401 11522763 PMC131040

[B44] Mazziotta J. C. Toga A. W. Evans A. Fox P. Lancaster J. (1995). A probabilistic atlas of the human brain: theory and rationale for its development: the international consortium for brain mapping (ICBM). NeuroImage 2, 89–101. 9343592 10.1006/nimg.1995.1012

[B45] McGonigle J. E. Mirmehdi M. Malizia A. L. (2010). Empirical mode decomposition in data-driven fMRI analysis, in Proceedings of the IEEE Workshop on Brain Decoding: Pattern Recognition Challenges in Neuroimaging (Istanbul), 25–28. 10.1109/WBD.2010.14

[B46] Moelker A. Pattynama P. M. (2003). Acoustic noise concerns in functional magnetic resonance imaging. Hum. Brain Mapp. 20, 123–141. 10.1002/hbm.10134 14601139 PMC6872037

[B47] Moeller S. Yacoub E. Olman C. A. Auerbach E. Strupp J. Harel N.. (2010). Multiband multislice GE-EPI at 7 tesla, with 16-fold acceleration using partial parallel imaging with application to high spatial and temporal whole-brain fMRI. Magn. Reson. Med. 63, 1144–1153. 10.1002/mrm.22361 20432285 PMC2906244

[B48] Murphy K. Birn R. M. Handwerker D. A. Jones T. B. Bandettini P. A. (2009). The impact of global signal regression on resting state correlations: are anti-correlated networks introduced? NeuroImage 44, 893–905. 10.1016/j.neuroimage.2008.09.036 18976716 PMC2750906

[B49] Murphy K. Fox M. D. (2017). Towards a consensus regarding global signal regression for resting state functional connectivity MRI. NeuroImage 154, 169–173. 10.1016/j.neuroimage.2016.11.052 27888059 PMC5489207

[B50] Niazy R. K. Xie J. Miller K. Beckmann C. F. Smith S. M. (2011). Spectral characteristics of resting state networks. Prog. Brain Res. 193, 259–276. 10.1016/B978-0-444-53839-0.00017-X 21854968

[B51] Penttonen M. Buzsáki G. (2003). Natural logarithmic relationship between brain oscillators. Thalamus Relat. Syst. 2, 145–152. 10.1017/S1472928803000074

[B52] Power J. D. Plitt M. Laumann T. O. Martin A. (2017). Sources and implications of whole-brain fMRI signals in humans. NeuroImage 146, 609–625. 10.1016/j.neuroimage.2016.09.038 27751941 PMC5321814

[B53] Qian L. Zhang Y. Zheng L. Shang Y. Gao J.-H. Liu Y. (2015). Frequency dependent topological patterns of resting-state brain networks. PLoS ONE 10:e124681. 10.1371/journal.pone.0124681 25927525 PMC4415801

[B54] Ravicz M. E. Melcher J. R. Kiang N. Y.-S. (2000). Acoustic noise during functional magnetic resonance imaging. J. Acoust. Soc. Am. 108, 1683–1696. 10.1121/1.1310190 11051496 PMC2270941

[B55] Riffi J. Adnane M. M. Abbad A. Tairi H. (2014). 3D extension of the fast and adaptive bidimensional empirical mode decomposition. Multidim. Syst. Signal Process. 26, 823–834. 10.1007/s11045-014-0283-6

[B56] Riffi J. Mahraz A. M. Tairi H. (2013). Medical image registration based on fast and adaptive bidimensional empirical mode decomposition. IET Image Process. 7, 567–574. 10.1049/iet-ipr.2012.0034

[B57] Rubinov M. Sporns O. (2009). Complex network measures of brain connectivity: uses and interpretations. NeuroImage 52, 1059–1069. 10.1016/j.neuroimage.2009.10.003 19819337

[B58] Saad Z. S. Gotts S. J. Murphy K. Chen G. Jo H. J. Martin A.. (2012). Trouble at rest: how correlation patterns and group differences become distorted after global signal regression. Brain Connect. 2, 25–32. 10.1089/brain.2012.0080 22432927 PMC3484684

[B59] Sharot T. Delgado M. R. Phelps E. A. (2005). How emotion enhances the feeling of remembering. Nat. Neurosci. 7, 1376–1380. 10.1038/nn1353 15558065

[B60] Shmuel A. Leopold D. A. (2008). Neuronal correlates of spontaneous fluctuations in fMRI signals in monkey visual cortex: implications for functional connectivity at rest. Hum. Brain Mapp. 29, 751–761. 10.1002/hbm.20580 18465799 PMC6870786

[B61] Shmueli K. van Gelderen P. de Zwart J. A. Horovitz S. G. Fukunaga M. Jansma J. M.. (2007). Low-frequency fluctuations in the cardiac rate as a source of variance in the resting-state fMRI bold signal. NeuroImage 38, 306–320. 10.1016/j.neuroimage.2007.07.037 17869543 PMC2128785

[B62] Song X. Hu X. Zhou S. Xu Y. Zhang Y. Yuan Y.. (2015). Association of specific frequency bands of functional MRI signal oscillations with motor syptoms and depression in Parkinson's disease. Sci. Rep. 5:16376. 10.1038/srep16376 26574049 PMC4648086

[B63] Song X. Zhang Y. Liu Y. (2014). Frequency specificity of regional homogeneity in the resting-state human brain. PLoS ONE 9:e86818. 10.1371/journal.pone.0086818 24466256 PMC3900644

[B64] Tagliazucchi E. Balenzuela P. Fraiman D. Chialvo D. (2012). Criticality in large-scale brain fMRI dynamics unveiled by a novel point process analysis. Front. Physiol. 3:15. 10.3389/fphys.2012.00015 22347863 PMC3274757

[B65] Tagliazucchi E. Balenzuela P. Fraiman D. Montoya P. Chialvo D. R. (2011). Spontaneous bold event triggered averages for estimating functional connectivity at resting state. Neurosci. Lett. 488, 158–163. 10.1016/j.neulet.2010.11.020 21078369 PMC3014405

[B66] Tong Y. Frederick B. (2014). Studying the spatial distribution of physiological effects on bold signals using ultrafast fMRI. Front. Hum. Neurosci. 8:196. 10.3389/fnhum.2014.00196 24744722 PMC3978361

[B67] Torres M. E. Colominas M. A. Schlotthauer G. Flandrin P. (2011). A complete ensemble empirical mode decomposition with adaptive noise, in Proceedings of the 36th IEEE International Conference on Acoustics, Speech and Signal Process, ICASSP 2011 (Prague), 4144–4147. 10.1109/ICASSP.2011.5947265

[B68] Turchi J. Chang C. Ye F. Q. Russ B. E. Yu D. K. Cortes C. R.. (2018). The basal forebrain regulates global resting-state fMRI fluctuations. Neuron 97, 940–952.e4. 10.1016/j.neuron.2018.01.032 29398365 PMC5823771

[B69] Tzourio-Mazoyer N. Landeau B. Papathanassiou D. Crivello F. Etard O. Delcroix N.. (2002). Automated anatomical labeling of activations in SPM using a macroscopic anatomical parcellation of the MNI MRI single-subject brain. NeuroImage 15, 273–289. 10.1006/nimg.2001.0978 11771995

[B70] Weissenbacher A. Kasess C. Gerstl F. Lanzenberger R. Moser E. Windischberger C. (2009). Correlations and anticorrelations in resting-state functional connectivity MRI: a quantitative comparison of preprocessing strategies. NeuroImage 47, 1408–1416. 10.1016/j.neuroimage.2009.05.005 19442749

[B71] Wu Z. Huang N. Chen X. (2009). The multi-dimensional ensemble empirical mode decomposition method. Adv. Adapt. Data Anal. 1, 339–372. 10.1142/S1793536909000187

[B72] Wu Z. Huang N. E. (2009). Ensemble empirical mode decomposition: a noise-assisted data analysis method. Adv. Adapt. Data Anal. 1, 1–41. 10.1142/S1793536909000047

[B73] Yeh J. R. Shieh J. S. Huang N. E. (2010). Complementary ensemble empirical mode decomposition: a novel noise enhanced data analysis method. Adv. Adapt. Data Anal. 2, 135–156. 10.1142/S1793536910000422

[B74] Yves M. (1993). Wavelets-algorithms and applications. Wavelets-Algorith. Appl. Soc. Indus. Appl. Math. Transl. 36, 526–528.

[B75] Zarahn E. Aguirre G. D'Esposito M. (1997). Empirical analyses of bold fMRI statistics. I. spatially unsmoothed data collected under null-hypothesis conditions. NeuroImage 5, 179–197. 10.1006/nimg.1997.0263 9345548

[B76] Zhan Z. Xu L. Zuo T. Xie D. Zhang J. Yao L.. (2014). The contribution of different frequency bands of fMRI data to the correlation with EEG alpha rhythm. Brain Res. 1543, 235–243. 10.1016/j.brainres.2013.11.016 24275197

[B77] Zheng T. Cai M. Jiang T. (2010). A novel approach to activation detection in fmri based on empirical mode decomposition. J. Integr. Neurosci. 9, 407–427. 10.1142/S021963521000255X 21213412

